# Prior beliefs and the interpretation of scientific results

**DOI:** 10.1098/rsos.231613

**Published:** 2023-12-20

**Authors:** Ami Eidels

**Affiliations:** School of Psychological Science, The University of Newcastle, Callaghan, New South Wales, Australia

**Keywords:** metascience, research methods, credibility, transparency, replication, real-time procedures

## Abstract

How do prior beliefs affect the interpretation of scientific results? I discuss a hypothetical scenario where researchers publish results that could either support a theory they believe in, or refute that theory, and ask if the two instances carry the same weight. More colloquially, I ask if we should overweigh scientific results supporting a given theory and reported by a researcher, or a team, that initially did not support that theory. I illustrate the challenge using two examples from psychology: evidence accumulation models, and extra sensory perception.

## 1. Introduction

How do prior beliefs affect the interpretation of scientific results? Consider the following hypothetical scenario: Alexander and Beatrice are two scientists interested in the same phenomenon, but with different beliefs about what they think is the best account, or theory underlying that phenomenon. Over the past years, Alex has published a dozen papers supporting theory A, and denouncing theory B. Bea and her laboratory members published a similar number of papers in favour of B, and against A. Recently, they each completed (separately) a new, critical experiment supporting theory A. To be convinced, would Bea require a larger amount of evidence than Alex? Then, when the two papers are published, how should the scientific community endorse the outcomes? Suppose Bea, a long-time proponent of theory B, had now published results that support theory A and contradict much of her prior work; should we overweight her new outcome and consider them more informative, compared with Alex, who had already articulated his support for theory A?

If we assume that the studies in Alex and Bea's laboratories were conducted with the same rigour, it seems unfair to ascribe greater importance to the same results coming from one laboratory and not the other. If we limit our interpretation to the data, and outcomes, and ignore the historical context, objective interpretation dictates identical results should be deemed equally informative. After all, the scientific method is commonly understood as the process of objectively establishing facts based on experimental testing (although philosophers of science, and scientists themselves, still argue about the importance of objectivity and whether it is attainable; e.g. [1]; see also [2]). However, there is something compelling in the fact Bea, who had advocated for theory B her entire career, is now reporting evidence to support theory A. Should readers of her results take this into consideration, and if so, how?

Such scenarios are not only hypothetical. In the next section, I present two scenarios in experimental psychology that help illustrate the challenges to scientific interpretation posed by prior beliefs. The first example concerns competing models of decision making; the second example concerns Bem's [3] experiment on extrasensory perception and the multi-laboratory replication attempt recently published in the Royal Society [4]. These examples propel a broader discussion about the way our prior beliefs and expectations affect our interpretation of scientific results, and possibly the amount of the evidence we require to update our beliefs.^1^

## 2. Example 1: evidence accumulation models

Evidence accumulation models describe human decisions and the time course of those decisions. These models propose that evidence accumulates for decision alternatives at some rate, until the evidence for one alternative reaches some threshold that triggers a decision [6]. I focus on two successful instances, the Wiener diffusion process and the linear ballistic accumulator (LBA), but many others exist (e.g. [7–11]).

The Wiener diffusion model, championed by Ratcliff and co-workers [12,13] describes the decision between two alternatives as a noisy process, where the state of the system—marking the momentary evidence in favour of some response, or its alternative—hovers up and down between two bounds until one of the bounds is reached and a decision is made. The LBA [14] differs in several important ways, and the most interesting is perhaps its ballistic nature: the accumulation process per any single decision is deterministic, not noisy. Developers of cognitive models traditionally collect empirical data and fit it using their own model to demonstrate its scientific utility, and perhaps supremacy over other candidate models. Indeed, both Ratcliff and colleagues, and well as Brown, Heathcote and their colleagues, published scores of papers demonstrating how their model fits data. The question then arises: if someone were to fit the two models to some dataset(s), what amount of evidence would Ratcliff be seeking to be convinced an alternate model, not their progeny, provides the best explanation for the data?^2^ Likewise, would Brown and Heathcote be requiring more evidence (more participants, more statistical power, larger effect size, more replications, etc.) against the LBA to prove it wrong, than they would for the Wiener diffusion model? Also, importantly, is there an objective way to quantify this expectation?

## 3. Example 2: extra sensory perception

In 2011, Daryl Bem published a series of experiments supporting extra sensory perception (ESP). The article, ‘Feeling the future: experimental evidence for anomalous retroactive influences on cognition and affect’, challenged common scientific conceptions and became an instant hit, if one is allowed to refer this way to a scientific paper. In Bem's experiment 1, participants in the laboratory (*n* = 100) were presented with two curtains on computer screen and had to guess which curtain hides an erotic picture. If participants were to simply guess at random, argued Bem, they should identify the position of the hidden erotic picture at chance (50%). Instead, participants correctly identified the future location of the erotic pictures at a rate of 53.1%, which was statistically better than chance. As can be expected, this reported ability to predict the future ignited immediate interest and, since 2011, there had been many replication attempts. As with the hypothetical example and the decision-making models' example above, one could ask whether we are playing on an even ground when it comes to evidence in favour or against ESP. That is, would a reader sceptic of ESP require more evidence in favour of ESP to change their mind, than they would to maintain their present anti-ESP belief.

## 4. Kekecs *et al.* [4] replication, and the quantification of prior beliefs

Arguably the most comprehensive replication attempt to-date is the *Royal Society Open Science* multi-laboratory effort by Kekecs *et al.* [4]. The authors of this paper had replicated Bem's experiment 1 across 10 different sites (laboratories), with 2115 participants yielding a total of 37 836 trials. The rate of successful guesses was 49.89%, which did not replicate Ben's better-than-chance rate. Among the many methodological innovations, aimed at improving the rigour of testing and analyses, the authors surveyed the prior beliefs of the research-team members. Specifically, all the key investigators involved in the replication study completed the Australian Sheep Goat scale, a questionnaire devised to assess belief in the paranormal [15]. Readers are invited to examine again table 1 in Kekecs *et al.*, and speculate about possible relationships between the researchers' beliefs (as recorded by the Sheep Goat scale), and the replication outcomes.

Directly asking for one's beliefs prior to data collection is already an important step. However, self-reports are subjective and often biased. An alternate approach, that indirectly probes their beliefs, could be to quantify the body of work produced by Alex and Bea. The more evidence they have found (and the more papers they have published) in favour of, say, theory A, the higher the weight of their prior belief in A. Such a counting process could be proved an excruciating exercise at present, but perhaps not in the future. Futuristic artificial intelligence (AI) tools could scan the body of relevant literature, and quantify—for each researcher—the extent of their prior belief in some theory based of what they have reported to-date. I have asked chatGPT ‘how strong is Ratcliff's belief in the diffusion model?’, and ‘what proportion of Ratcliff's papers support the diffusion model?‘. The answer was vague (ChatGPT 2023, A. Eidels 2023, personal communication), and yet some proportion index, contrasting the number of articles in favour of A, over the papers in favour of B, may offer a window—albeit crude—to researchers' prior beliefs. Using either self-reports, as in Kekecs *et al.*, or some futuristic AI index, a new question then arises: whether and how should the reader take information about researchers' prior beliefs into account when interpreting scientific results. I leave this question to future discussions. A related question I leave the reader to grapple with is this: are all articles equal, or is our belief affected (and should it be affected) by contextual factors such as the perceived quality of the outlet in which it is published, or the type of the article. For example, given two articles reporting contradictory findings, should scientists, and in the future the AI, overweigh evidence from a *Registered Report*, such as Kekecs *et al.*, over its non-registered competitor (see [16] for a comprehensive discussion); should we put a premium on the one published in the ‘better’ journal; and lastly, to what extent do we already take these considerations into account.

## Data Availability

This article has no additional data.

## Footnotes

1 Proponents of Bayesian reasoning can see these examples are not unlike Bayesian prior updating [5]. In Bayesian statistics, *a priori* knowledge is expressed as a prior distribution, marking our expectations. This knowledge is then combined with new observations (data) to form the posterior distribution, which is our updated belief. Critically for our example, two individuals with different priors could end up with different posterior beliefs, even if they observed *exactly* the same data.

2 Such inference typically considers the goodness-of-fit (how well the model captures trends in the data), as well the complexity of the model (everything equal, simpler models would be favoured).
